# Introduction from the Editors

**DOI:** 10.1186/1710-1492-7-S1-I1

**Published:** 2011-11-10

**Authors:** Harold L Kim, Richard Warrington, Wade Watson

**Affiliations:** 1 McMaster University, Hamilton, Ontario, Canada; 2 University of Western Ontario, London, Ontario, Canada; 3 University of Manitoba, Winnipeg, Manitoba, Canada; 4 Dalhousie University, Division of Allergy, IWK Health Centre, Halifax, Nova Scotia, Canada

## 

Recent evidence suggests that the prevalence of various allergic conditions such as allergic rhinitis, food allergy and eosinophilic esophagitis (EoE) is on the rise [1-3]. Over the last decade, there have been significant advances in our current understanding of these as well as other common allergic and immunological diseases. The purpose of this supplement entitled, *A Practical Guide for Allergy and Immunology in Canada*, is to provide medical students, medical residents, primary-care practitioners and other healthcare professionals with a comprehensive, yet easy-to-follow, series of articles on all of the common conditions we deal with in the field of allergy and immunology.

The first article provides a basic introduction to the main components and function of the immune system and its role in both health and disease, and also serves as a background to the immunopathological disorders discussed in the remainder of this supplement, including asthma, allergic rhinitis, atopic dermatitis (AD), anaphylaxis, food allergy, EoE, urticaria, angioedema, drug allergy and primary immunodeficiency disorders (PIDs). Asthma remains the most common chronic respiratory disease in Canada [4], and despite significant improvement in the diagnosis and management of this disorder, the majority of Canadians with asthma remain poorly controlled [5]. Allergic rhinitis frequently coexists with asthma [6], and it is often a long-standing condition that goes undetected in the primary-care setting. AD is one of the most common skin disorders in children [7], and is often the initial step in the “atopic march” (the sequential development of allergic disease manifestations during early childhood), which leads to asthma and/or allergic rhinitis in the majority of afflicted patients [8]. Often referred to as “asthma of the esophagus”, EoE is an atopic inflammatory disease of the esophagus that has also become increasingly recognized over the last decade. This supplement provides an overview of the epidemiology and pathophysiology of these common allergic conditions as well as practical strategies for their diagnosis and management.

Allergen-specific immunotherapy is the only potentially disease-modifying therapy for allergic disease and has been proven to be effective for the treatment of allergic rhinitis/conjunctivitis, allergic asthma and stinging insect hypersensitivity. However, despite its proven efficacy, it is frequently underutilized in Canada. In this supplement, the authors review the indications and contraindications, patient selection criteria, and the administration, safety and efficacy of allergen-specific immunotherapy.

Anaphylaxis is an acute, potentially fatal systemic reaction that requires prompt recognition and treatment; however, both patients and healthcare professionals often fail to recognize and diagnose its early signs and symptoms. The accurate diagnosis and appropriate management of food allergy is also critical since accidental exposure to even minute quantities of the “culprit” food may result in anaphylaxis. In this supplement, the authors discuss the causes, clinical features, diagnosis and management of anaphylaxis and food allergy. The authors also review the pathophysiology, diagnosis and treatment of urticaria (hives) and the work-up and management of isolated angioedema (swelling that occurs beneath the skin), which vary considerably from that of angioedema that occurs in the presence of urticaria. Although isolated angioedema is often self-limited, laryngeal involvement can lead to fatal asphyxiation in some cases; therefore, prompt recognition and management are imperative.

Drug allergy encompasses a spectrum of immunologically-mediated hypersensitivity reactions that not only affect patient quality of life, but that may also lead to delayed treatment, unnecessary investigations, and even mortality. In this supplement, the authors examine the most common drug-induced allergic reactions, such allergies to penicillin, sulfonamides, cephalosporins, radiocontrast media, local anesthetics, general anesthetics, acetylsalicylic acid (ASA) and non-steroidal anti-inflammatory drugs (NSAIDs).

The authors also provide a detailed overview of the major categories of PIDs (a heterogeneous group disorders that result from defects in immune system development and/or function). Although the clinical manifestations of PIDs are highly variable, most disorders involve an increased susceptibility to infection. In fact, many PIDs present as “routine” respiratory infections and, therefore, maygo undetected in the primary-care setting.

For each of the above-mentioned articles, key take-home messages are summarized for quick reference and, where applicable, easy-to-follow flow charts, tables and algorithms are provided to assist clinicians in the identification, diagnosis and treatment of these common allergic diseases. We are confident that readers will not only find this supplement educational and informative, but that it will also provide clinicians with a solid base of knowledge and skills in allergy and immunology which they can then incorporate into their respective clinical practices to help improve the care and management of patients with allergic disease. It is also our hope that this supplement will spark further interest in these conditions and in our specialty.

Finally, we would like to thank all of the authors who set aside time from their numerous commitments to write and review these informative articles, the peer reviewers for providing their highly-valued feedback, and the Canadian Society of Asthma and Clinical Immunology and other sponsors who provided the support needed for the development of this important educational initiative.

We sincerely hope you enjoy this supplement!

Harold Kim, MD, FRCPC Richard Warrington, MB, BS, PhD, FRCPC Wade Watson, MD, MEd, FRCPC.

## Competing interests

Dr. Harold Kim is the past president of the Canadian Network for Respiratory Care and co-chief editor of *Allergy*, *Asthma &Clinical Immunology*. He has received consulting fees and honoraria for continuing education from AstraZeneca, GlaxoSmithKline, Graceway Pharmaceuticals, King Pharma, Merck Frosst, Novartis, and Nycomed.

Dr. Richard Warrington is the past president of the Canadian Society of Allergy & Clinical Immunology and Editor-in-Chief of *Allergy*, *Asthma & Clinical Immunology.* He has received consulting fees and honoraria from Nycomed, CSL Behring and Talecris.

Dr. Wade Watson is a co-chief editor of *Allergy*, *Asthma & Clinical Immunology.* He has received consulting fees and honoraria for continuing education from AstraZeneca, GlaxoSmithKline, King Pharma, Merck Frosst, and Nycomed.
